# Dental Implant Abutment Screw Loss: Presentation of 10 Cases

**DOI:** 10.3390/jfb15040096

**Published:** 2024-04-09

**Authors:** Maryam Soleimani, Jarosław Żmudzki, Wojciech Pakieła, Anna Jaśkowska, Kornel Krasny

**Affiliations:** 1 Department of Engineering Materials and Biomaterials, Faculty of Mechanical Engineering, Silesian University of Technology, 18a Konarskiego Str., 41-100 Gliwice, Poland; maryam.soleimani@polsl.pl (M.S.); wojciech.pakiela@polsl.pl (W.P.); 2 Doctoral School, Silesian University of Technology, 2A Akademicka Str., 44-100 Gliwice, Poland; 3 Anident Dental Clinic, 12 Belgradzka Str., 02-793 Warszawa, Polandkornel.krasny@op.pl (K.K.)

**Keywords:** dental implant abutment screw, titanium alloy, titanium nitride coating, failure, fracture, loosening, unscrewing, pitting corrosion

## Abstract

Re-tightening the loosened dental implant abutment screw is an accepted procedure, however the evidence that such screw will hold sufficiently is weak. The purpose of this study was material analysis of lost dental implant abutment screws made of the TiAlV alloy from various manufacturers, which became lost due to unscrewing or damaged when checking if unscrewed; undamaged screws could be safely re-tightened. Among 13 failed screws retrieved from 10 cases, 10 screws were removed due to untightening and 3 were broken but without mechanical damage at the threads. Advanced corrosion was found on nine screws after 2 years of working time on all surfaces, also not mechanically loaded. Sediments observed especially in the thread area did not affect the corrosion process because of no pit densification around sediments. Pitting corrosion visible in all long-used screws raises the question of whether the screws should be replaced after a certain period during service, even if they are well-tightened. This requires further research on the influence of the degree of corrosion on the loss of the load-bearing ability of the screw.

## 1. Introduction

In dental implants, various types of failures may occur at the implant–abutment connection [1,2]. The abutment and screw are mainly made with titanium-vanadium-aluminum alloy (Ti6Al4V). There are many studies on load transfer onto abutment and implant–abutment connection, and the abutment screw itself between the head and screw thread [3,4,5]. Tests of contact stresses between the abutment and the implant reveal fretting and wear phenomena of the less harder pure titanium implant surface, but also tribo-chemical processes in the alloy [6,7,8].

Pitting corrosion is suggested to be the mechanism of the alloy degradation [9]. However, there is weak clinical study evidence for this, especially in the case of abutment screws which are not in direct contact with tissues and the oral environment. Available literature shows that re-tightening the mounting screw is an accepted procedure [10]. Routine screw replacement is not recommended, but routine screw-tightening assessment is recommended to minimize more serious complications. It seems reasonable to ask whether the unscrewed abutment screw is still a valid screw and can be reused to mount a prosthetic work.

The aim of this work was a material analysis of dental implant abutment screws made of the TiAlV alloy from various manufacturers which became lost due to unscrewing or damage. 

## 2. Materials

### 2.1. Clinical Study

On average, 300 implants are implanted annually at the ANIDENT Dental Clinic, and 645 implants were inspected from the period between 1 January 2023 and 1 June 2023. These were cases of our own and external patients. Among them, there were 45 loosened screws, including 7 broken ones. Among the 45 screws removed, 36 were single crowns and 9 were screws in prosthetic bridges (implants connected by a superstructure). Only 3 screws were from the anterior segment, but all of them were connected by a bridge to the posterior teeth, and 42 screws were from posterior crowns. Patients reported feeling the mobility of a single or multi-point denture. Patients who presented with a loose crown reported biting, for example, an eggshell, nut, bone, or salt crystal. They claimed that they felt this event, but without any consequences, and only after a few weeks did they notice the crown loosening. Interestingly, they claimed that they felt the loosening on soft foods such as a roll. In cases where the screws broke, it was easy to unscrew them. In other cases, the loose screws were removed also without any problems, disinfected, and sent for material assessment. A total of 13 screws from 10 cases (CsNo) were selected as representative for material investigation. Table 1 shows the characteristics of these clinical cases.

### 2.2. SEM 

Analysis of the implant screw surfaces were made using a Supra 35 scanning electron microscope from Zeiss (Jena, Germany). Observations were made in both SE and InLens modes. The analysis of the chemical composition in micro-areas and the element distribution maps were performed using an EDS scattered X-ray detector from Thermo Fisher Scientific (Waltham, MA, USA) at an accelerating voltage in the range from 10 to 20 keV and the required work distance of 14 mm.

### 2.3. Light Microscopy

A digital microscope (Leica DVM6 A, Wetzlar, Germany) with image sensor 1/2.3″ CMOS 3664 × 2748 pixel and LED light source software-controlled was used. Images with resolution from 2MP (1600 × 1200) to 10MP (3648 × 2736) were snaped with LAS X software 5.0.3. Automated motorized focus drive allows 3D imaging with resolution of 0.25 µm in vertical direction. Screws were investigated with two lenses (PlanAPO FOV 43.75, working distance: 60 mm, max. magnification: 190:1, max. resolution: 415 lp/mm; PlanAPO FOV 12.55, working distance: 33 mm, max. magnification: 675:1, max. resolution: 1073 lp/mm)

Metallographic examinations of the specimens were performed on the implant using 2 broken screws, CsNo2 and CsNo10, using a light microscope (Zeiss Axio VertA1 MET Brightfield/Darkfield Metallurgical Microscope, Oberkochen, Germany). Cross-sections were standardly ground, polished, and etched with Kroll’s reagent (Sigma Aldrich, Darmstadt, Germany). Grinding and pit searching were repeated, and the deepest pits were documented.

### 2.4. Surface Roughness

The roughness measurement was made with using mechanical contact profilometer (Taylor Hobson, Leicester, United Kingdom) on the shank along the screw axis. Due to the impossibility of measuring perfectly on the axis, the curvature of the cylinder was superimposed on the roughness measurement.

## 3. Results

### 3.1. Clinical Observations

**Figure 1:**
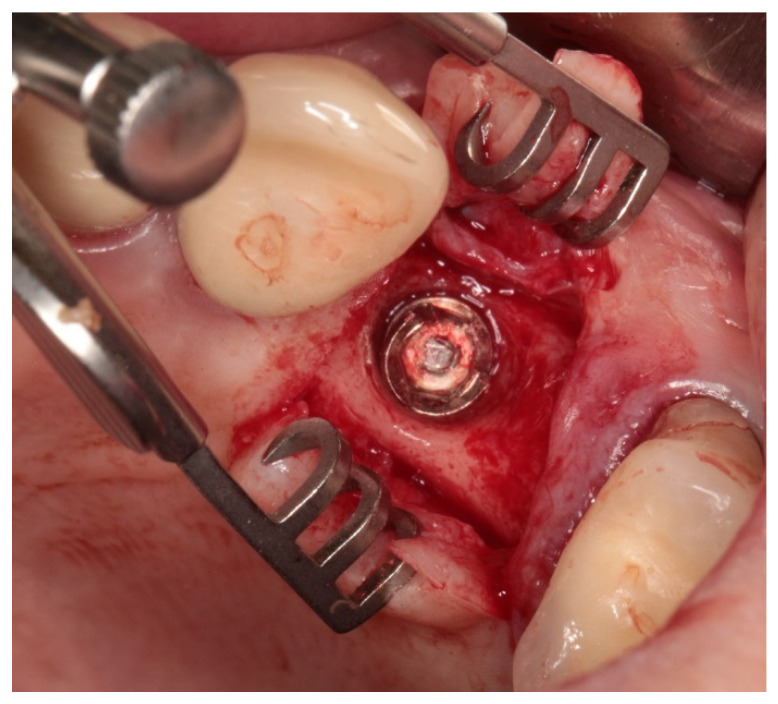
Service of broken screw exemplary case.

**Figure 2:**
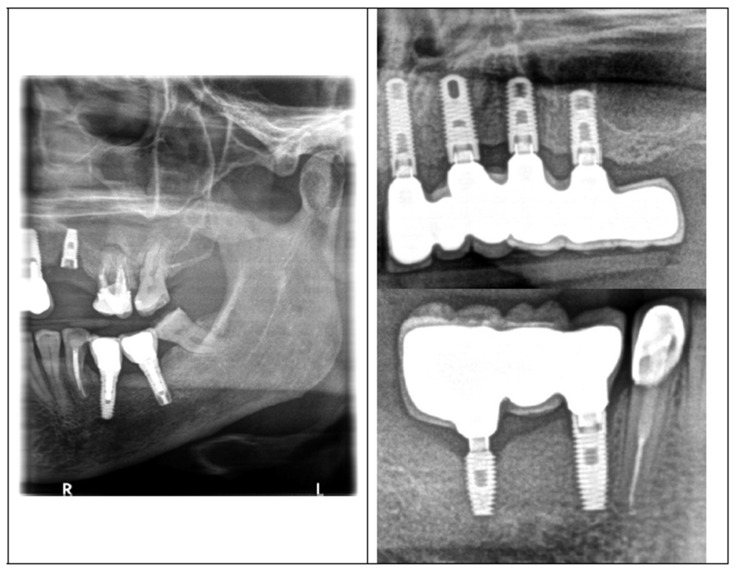
X-ray exemplary imaging of the screw failures: left X-ray, loose/broken screw in the upper left premolar CsNo8; upper-right X-ray, broken screws in implants 21 and 23 and loosened screws in implants 24 and 25 CsNo2; lower-right X-ray, broken hex in implant 46, both screws in 44 and 46 were loose in CSNo7.

Figure 1 shows an example of a broken screw, while Figure 2 shows X-ray exemplary images of the failures. The case histories were known and the duration of screw use is given in Table 2.

The analysis showed that single crowns were more likely to unscrew than those connected in prosthetic bridges. This seems logical, as when biting, there will be an additional rotational force on the chewing surface of the tooth. So far, we have not observed any unscrewing of the crown or bridge installed in the anterior section. The problem most often concerned the posterior section—the molar area. Screws regardless of diameter became loose. The sets we use included implants with a diameter of 2.7 mm—used only in the anterior section due to very narrow ridges and small interdental space—we have not observed any breakage or unscrewing of them. All other available diameters, i.e., 3.7, 4.1, and 4.7 were loosened or broken in the side sections. The doctor performing the procedure has no influence on the type, shape, or method of screwing/stabilizing the prosthetic superstructure. All these features depend on the implant socket, the shape of the hex, the depth of the hex, the type of screw stabilizing the superstructure, and the shape, depth, and type of hex of the prosthetic superstructure/connector. All these elements are specified and prepared by the implant manufacturer and were the same in all these cases.

### 3.2. Material Analysis

**Figure 3:**
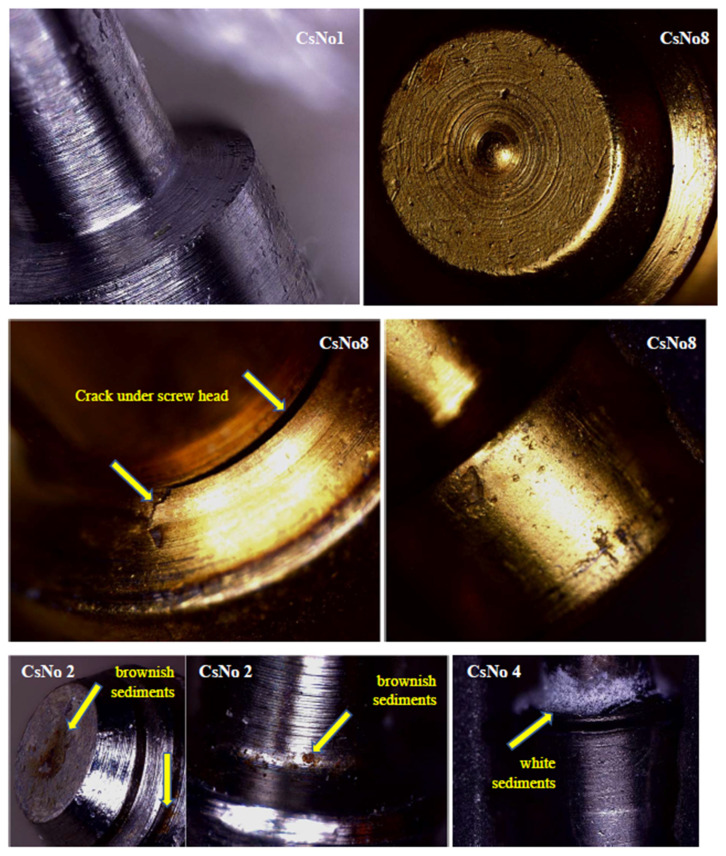
Pitting corrosion, crack on the shank below screw head, and brownish/white sediments shown in order: CsNo1, CsNo8 (TiNc), CsNo2, CsNo4.

**Figure 4:**
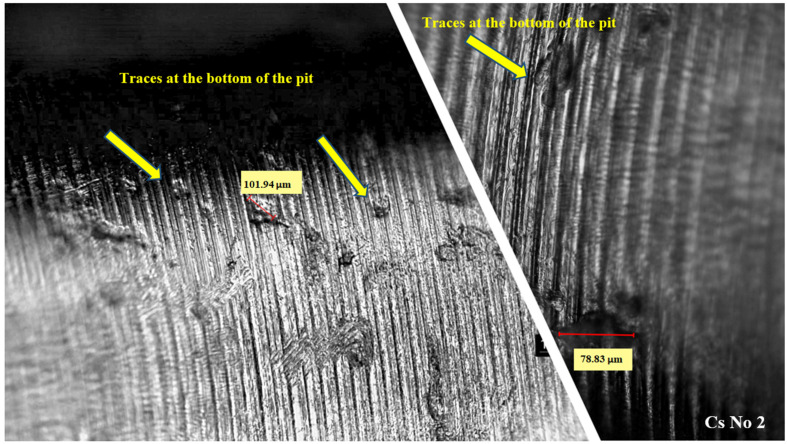
Pits on the shank of CsNo2 implant abutment screw and visible traces at the bottom of the pit.

Light microscopy revealed pitting corrosion in all screws, although in the one that had been in use for 1 year, initial pits were few and difficult to find. Figure 3 and Figure 4 show sample selected images for the cases: CsNo1, CsNo2, CsNo4, CsNo8. Pits were revealed on all surfaces and were not concentrated on the thread or screw head contact surfaces. There was also no density of pits around the sediments. No signs of wear or tribo-corrosion were revealed on the threads and screw heads. The threads showed no signs of plastic deformation, and the traces of mechanical machining were intact. Minor scratches and mechanical damage that were clearly caused by screwing and removing screws were not considered. Screw CsNo8 with a gold-colored titanium nitride coating (TiNc) was corroded after 2 years to at least the same extent as screws with a standard oxide coating that lasted in the oral cavity much longer. A screw with TiN coating had an extensive crack (but not a fracture) on the shank in the stress concentration area around the notch under the head.

**Figure 5:**
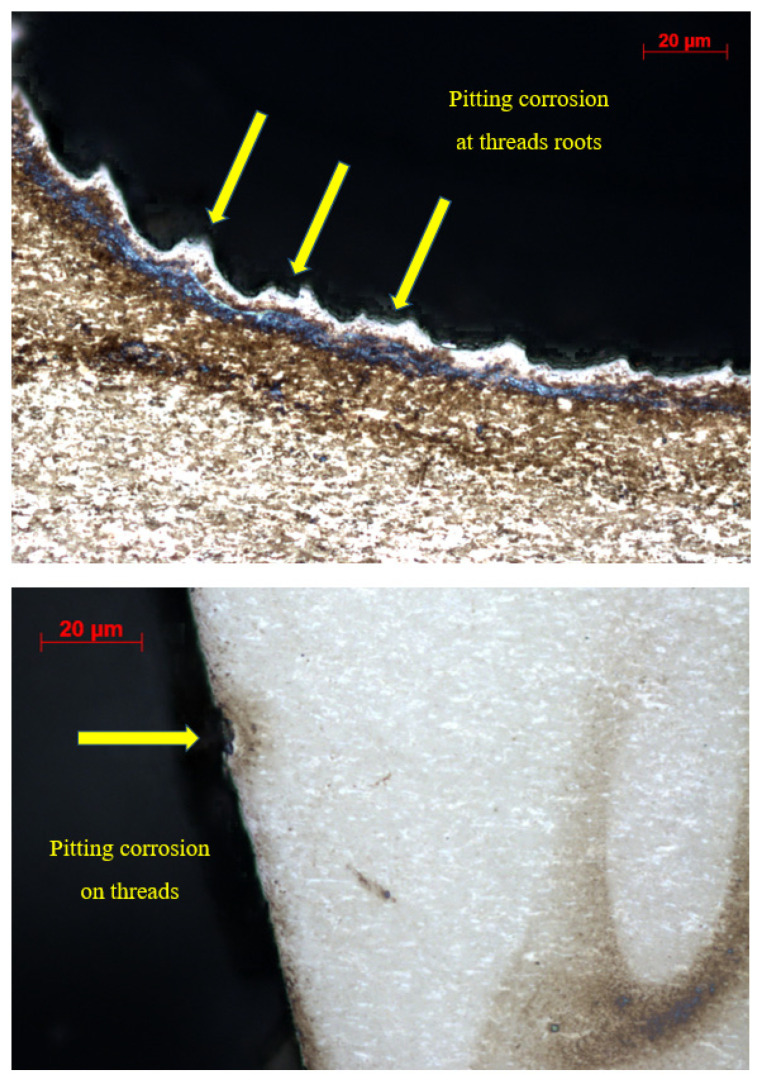
Examples of the deepest pitting corrosion found on the thread and the beginnings of dissolution of machining tips at thread root in cross-sectional micrographs of a CsNo2 implant screw.

**Figure 6:**
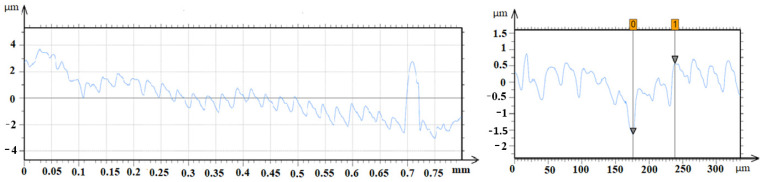
Roughness profile for implant screw CsNo2 with visible uniform traces of machining and clear dimensions of deposits, and an example of wide pitting corrosion with its depth. Region between 0 and 1 related to bottom and the edge of the pits respectively.

The selected screws for cross-sectional study had the most pits that appeared to be the deepest, however, repeated grinding and cross-sectional study allowed us to find the deepest pits as shown in Figure 5. In thread root, an example was found at the beginning of the dissolution of tips remaining after machining. The Figure 6 profile shows the roughness for the CsNo2 implant screw with visible uniform machining traces. Sediment heights were revealed, which are difficult to measure reliably on a microscope. An example of wide pitting corrosion was shown along with its depth, which turned out to be relatively shallow, as the depth relative to the adjacent tip only exceeded 2 microns. Due to the surprisingly shallow dimensions of the pits, we additionally looked at them under a microscope and, in fact, most of them were visible at the bottom of the remains of the machining grooves. The 3D measurement on the microscope also did not exceed a few microns, but due to the lower quantitative reliability of this technique, we chose a contact profilometer for presentation.

**Figure 7:**
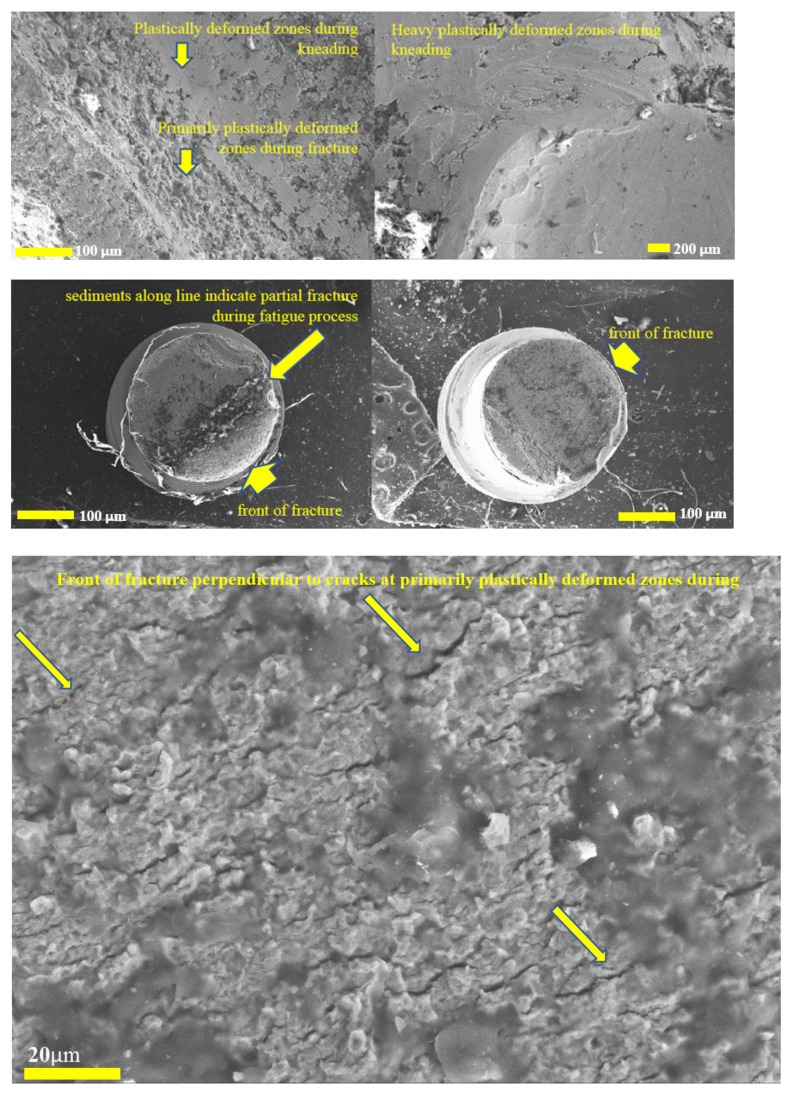
Fractures in the two broken screws among four implants which supported denture in case CsNo2.

The fractures in the broken screws in case CsNo2 as shown in Figure 7 were kneaded, which proves that still compression loads were transferred on the two remaining unbroken screws in the denture supported on 4 implants. At one of the fractures were visible sediments grouped along a line that appears to be a fatigue fracture. Fracture type was hard to estimate due to the destroyed fracture surface during kneading. Primarily, plastically deformed zones during fracture were mixed with those plastically destroyed during kneading, and the zone of fatigue initiation was not distinct. Numerous cracks were also visible in the areas of plastic rupture and ran parallel to the lines of the sediment cluster. The front of the fracture was indicated only on the basis of general shape and clear direction of cracks.

**Figure 8:**
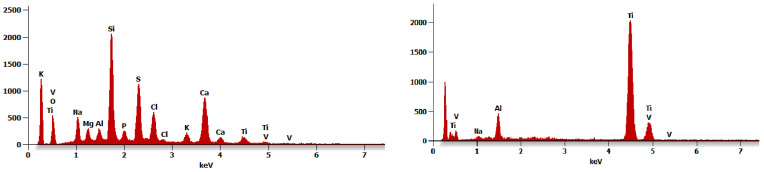
SEM EDS of brownish and white sediments.

The SEM EDS analysis of sediments presented in Figure 8 in places of signal, where different elements overlapped, were those that were least likely to be arbitrarily eliminated. Analysis showed that a brownish sediment mainly contained elements from food or toothpastes. The white sediment was rich in zinc.

**Figure 9:**
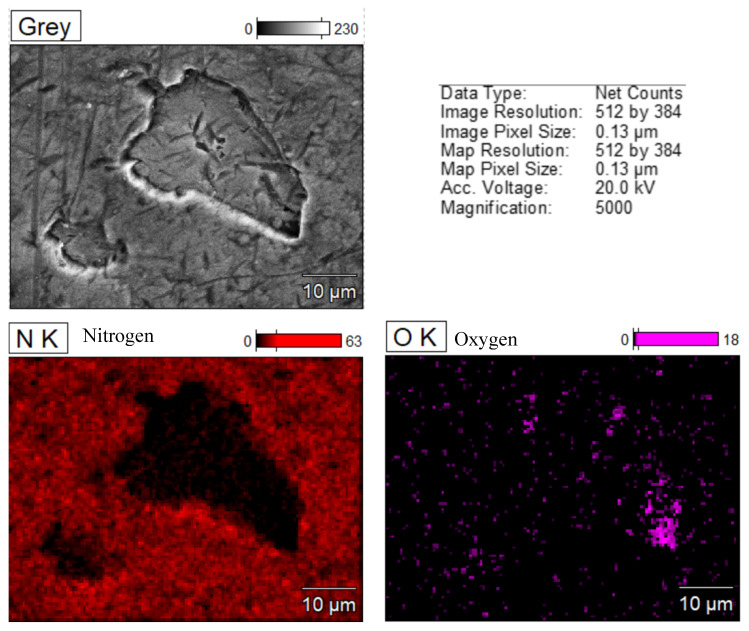

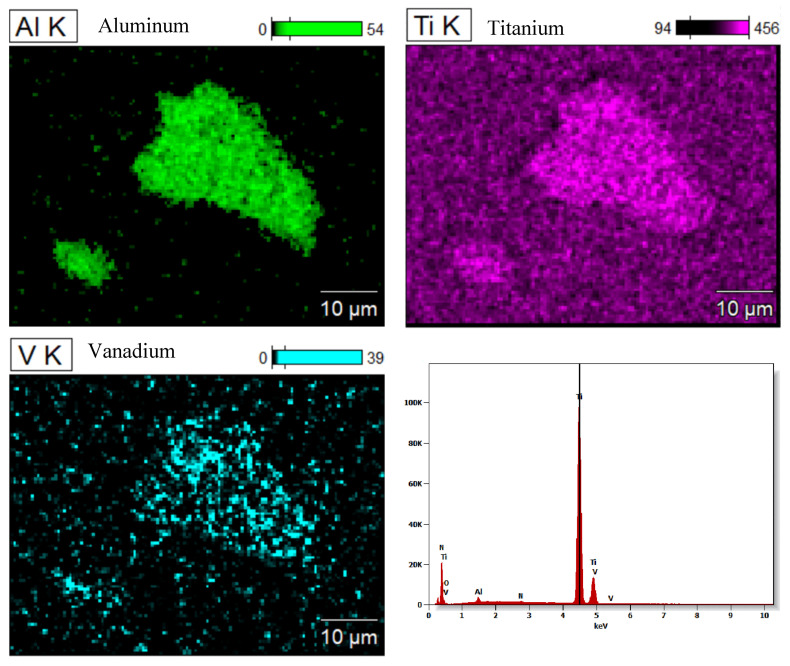
SEM EDS map around a corrosion pit on the CsNo8 implant screw with TiN coating.

Figure 9 shows an EDS map for a TiNc screw around a corrosion pit. The distribution of nitrogen shows its loss inside the pit, while more oxygen appears, which comes from the spontaneously formed titanium oxide. The exposure of the alloy substrate resulted in the appearance of a strong aluminum and vanadium signal, as well as a significant increase in the titanium signal.

## 4. Discussion

The analysis showed that among inspected implants all types of crowns are subject to unscrewing regardless of the implant diameter (3.7, 4.1, and 4.7 mm), implant abutment diameter, and used abutment screw. Analysis led to the conclusion that the implantation area (usually the lateral/distal section) had a greater impact on crown unscrewing rather than the implant diameter. 

In threaded connections, failure under cyclic loading is often caused by screw loosening and loss of preload due to micro-movements on the contacting thread surfaces. Especially with simultaneous activation of corrosion processes, micro-damages, fretting and wear, and plastic deformations of the thread will appear in heavily loaded areas, which result in the loss of preload. Among ten failed screws, seven screws were removed due to untightening and three were broken albeit without mechanical damage at the threads. Advanced corrosion was found on nine screws after 2 years of working time on all surfaces, also not mechanically loaded. A long crack on the one non-fractured screw was found located close to the notch on the shank under the screw head. Among three fractured screws, there was the one with the shortest working time of 1 year and there were difficulties in seeing a few initial shallow corrosion pits. The summary of our finding is that the existence of mechanical deformations or micro-damages on the threads has not been confirmed. 

The outcome observed in the in vivo result was unexpected, particularly because we anticipated the mechanical effects identified in in-vitro studies which were suggested as the mechanism for the decrease in screw preload and unscrewing [8,9,11,12,13]. However, our results were consistent with those showing pitting corrosion in this titanium alloy. In the work [9], on the basis of X-ray spectroscopy (EDS) of the Ti6Al4V implant removed from the patient due to inflammation, after one year it showed that the titanium and vanadium decreased on the surface and the content of aluminum slightly increased. The study is based on one implant and it is not known how it can be personally dependent. In the work, the initiation of micro cracks and the destruction of the implant under the influence of mechanical loads are suggested, however, there is no confirmation of the hypothesis. 

In the work [14], pitting attacks were observed in the five clinically retrieved implants, albeit pits, located mostly in the abutment surfaces over the implant edge. A crack nucleating inside a pit about 20 μm in size is documented. The implant bone interface discoloration is justified with oxidation of unprotected bulk titanium to the violet-colored trivalent ions (Ti 3+) and also in SEM/EDS analysis metal ion dissolution with depletion of the Ti oxide film. In the work, it is suggested that degradation is the combining effect of corrosion and mechanical loadings, because scratches, fretting, cracks, and surface delamination are observed. The discovery of pitting on many screws in mechanically weakly loaded and free from interfacial compression areas in our study shows that corrosion progresses inside the implant directly under the influence of the oral environment without contact with tissues or other materials. The screws came from different manufacturers, which allows us to conclude that this is not a material defect caused by the technological process of the selected manufacturer. We cannot confirm that the sediment was rich in elements of screw alloy. The signal from substrate metals may be an artifact and a thorough examination of the composition of the sediment requires its removal from the surface and examination separately from the substrate. We were able to evaluate signals from elements that are not present on the alloy substrate. The brownish sediment gave a signal of many elements that are commonly found in foods or toothpaste, while the white sediment gave a Zn-rich signal.

Corrosion of Ti6Al4V hip joint implants can occur in a tissue environment isolated from external factors present in the oral cavity [13]. However, some in vitro investigations [15,16,17,18] indicate that factors of the oral cavity may influence corrosion. Our observations lack a concentration of pits around the deposits, but this does not prove that there is no acceleration of corrosion by oral environmental factors that are found in saliva or biofilm. 

Measurements of the depth of the pits showed that they are very shallow and do not reach such depths as in in vitro tests. Our results also differ from the in vivo results of work [17] in which depth is assessed based on microscopic images from three hip implants. Based on our surface images, we were convinced of the great depth of the pits, but neither profilometer measurements nor cross-sectional imaging showed a depth greater than several microns. However, our samples were not exposed to friction, as in works [13,17], and did not show wear, cracks, or mechanical damages, which lead to failures in the oxide layer and a different mechanism of pitting corrosion. These depths were slightly greater than the roughness after the machining process, so their influence as a notch on fatigue strength seems to be very limited, although it cannot be ruled out, especially in the context of hydrogen corrosion demonstrated in the work [13], which we were unable to investigate due to our hardware limitations. However, in vivo pitting corrosion depth results indicate that in vitro corrosion simulation conditions may differ significantly from actual conditions for surfaces exposed only to corrosion without mechanical loads.

The limitation of the study was three manufacturers, and among the cases we had only one screw coated with TiN. The time-consuming statistical analysis of the number and dimensions of pits was abandoned, although determining the relationship between the growth of pits and the material working period may be useful in comparative studies between variable materials in the future. 

Our cases were limited to one similar design of implant–abutment connection. Incidence of screw loosening is related to many factors albeit among them only several factors depend on system design. Initial torque and clamped force loss are affected by torque magnitude, screw diameter, head and thread design, and implant–abutment connection design [17]. The compressive force that shortens the shank of a screw when it is greater than the preload in the shank can activate unscrewing. Hence, theoretically, the more flexible implant–abutment connection promotes screw loosening. The phenomena become complicated and compliance is not a clear determinant due to bending. On the tension side, the compliance prevents shortening of the screw. However, at the same time, the additional tension of the preloaded screw may cause it to break. In practice, screw loosening is a common mechanical complication with an incidence of 4.3–10% during the first year [17,18] and 12.7% in single crowns and 6.7% in fixed partial dentures [17]. In vitro, an overloading study suggests that the internal and external connection systems could not prevent screw loosening [19]. In the in vitro studies, on the basis of reverse torque value, this shows that the ITI system is the most stable and resistant to screw loosening compared to others [20,21]. In turn, according to work [22], one-piece abutments are more resistant to screw loosening than the two-piece. The use of compatible components with original implants showed significant micromovements when compared with the use of the same manufacturer part [23]. Other works present opposite results in that the non-original components are interchangeable and do not lead to screw loosening if manufacturing discrepancies are lower than 10 microns [5,24,25,26]. Manufacturers are not able to guarantee failure-free screws, so for each system, it is worth having proprietary tools for removing broken screws and, above all, using radiographic detection of the gap at the implant–abutment interface [27,28].

The limitation of these in vivo tests was the lack of an implant for testing, which, being softer with pure titanium, may be subject to wear and deformation, and we did not find any traces of this on the screws. Although ideal thread surfaces do not indicate such a scenario, it cannot be ruled out without testing the implants also at the implant–abutment interface, where wear leads to changes in the forces existing on the screw.

## 5. Conclusions

No clear signs of “fretting”, wear, or tribo-corrosion were found at the screw head and thread contact interfaces. However, numerous corrosion pits were found in many areas, including those not subject to mechanical loadings. Sediments observed especially in the thread area did not affect the corrosion process because of no pit densification around sediments.

The pitting corrosion visible in all long-used screws raises the question of whether the screws should be replaced after a certain period during service, even if they are well tightened. This requires further research on the influence of the degree of corrosion in in vivo conditions on the loss of fatigue strength of the screw.

## Figures and Tables

**Table 1:** Implant identification.

Case No.	Implant Side Anterior/Lateral	Denture Type	Implant Type/Abutment Dimensions and Manufacturer	Case Description
1	lateral	single crown 46	IRES 4.1 mm × 8 mm	The tested screw was used to attach a single crown in the lateral section 46 (mandibular first molar, right side) to an implant with a diameter of 4.1 × length of 8 mm. The implant was implanted in 2018, the crown was made in 2019, and the screw worked for four years. The patient did not come for regular check-ups and only came to the emergency room due to loosening of the crown.
2	anterior + lateral	bridge 6 pts. 21–26	ZIMMER 3.7 mm × 13 mm ZIMMER 4.1 mm × 11.5 mm ZIMMER 4.1 mm × 10 mm ZIMMER 4.1 mm × 10 mm	The four screws tested attached a six-point bridge to implants in sections 21–26 (front and lateral sections of the maxilla, left side). Implant in the incisor area with a diameter of 3.7 mm and a length of 13 mm, implant in the canine area with a diameter of 4.1 mm and a length of 11.5 mm, in the area of the first premolar with a diameter of 4.1 mm and a length of 10 mm, and last implant in the area of the molar tooth with a diameter 4 and 10 mm long. Implant placement in 2016. The prosthetic work was installed in 2020, the screws lasted for three years. The patient did not come for regular check-ups and started treatment in another office, which resulted in overloading the bridge on implants 21–26 and breaking two of the four implant-fixing screws in the premolar and molar area.
3	lateral	bridge 3 pts. 15–17	ZIMMER 3.7 mm × 11.5 mm ZIMMER 4.7 mm × 8 mm	The loosened screw came from a 3-point bridge based on two implants in the lateral part of the maxilla, right side 15–17. Implants in the area of the first premolar (14) with a diameter of 3.7 mm and a length of 11.5 mm (implanted in 2017) and the second one in the area of the first molar of the maxilla, right side (16) with a diameter of 4.7 mm and a length of 8 mm (implanted in 2018). The bridge was constructed in 2021, the screw operated for two years. The patient came for follow-up visits and the screw was tightened once.
4	lateral	single crown 14	ZIMMER 4.1 mm × 10 mm	The tested screw attached a single crown placed on the right side of the maxillary first premolar (14), lateral section. Implant with a diameter of 4.1 mm and a length of 10 mm. It was implanted in 2014. The prosthetic work was installed in 2015. The screw functioned for eight years. The patient did not attend regular follow-up visits. The screw did not come loose before.
5	lateral	single crown 37	ZIMMER 3.7 mm × 8 mm	The screw comes from a single crown on an implant placed near the second molar of the mandible on the left side (37), lateral section. The 3.7 mm diameter and 8 mm long implant was placed in 2015, and the single crown was placed in 2016. The screw lasted for six years. The patient came for follow-up visits sporadically and irregularly, and the screw was tightened twice.
6	lateral	single crown 36	ZIMMER 3.7 mm × 10 mm	The screw comes from a single crown on an implant placed near the first molar of the mandible, left side (36), lateral section. An implant with a diameter of 3.7 mm and a length of 10 mm was implanted in 2015. A single crown on the implant was placed in 2016. The screw functioned for six years. The patient came for follow-up visits sporadically, irregularly. The screw was tightened once.
7	lateral	bridge 3 pts. 44–46	IRES 3.75 mm × 11.5 mm IRES 3.75 mm × 10 mm	The screw tested comes from a 3-point bridge in the lateral section 44–46 mounted on two implants. Implants in the area of the mandibular first premolar (44) with a diameter of 3.75 mm and a length of 11.5 mm. And the second one in the area of the first molar of the mandible, right side (46), with a diameter of 3.75 and a length of 10 mm were implanted in 2018. The prosthetic work was installed in 2019. The tested screw functioned for four years. The patient did not come for regular check-ups, and the screws did not loosen earlier. The examination revealed, in addition to the loose screw, a fracture of the implant 46.
8	lateral	single crown 24	IRES 4.1 mm × 11.5 mm	The tested screw was used to attach a single crown to the implant in the area of the maxillary first premolar, left side (24), lateral section. Implant with a diameter of 4.1 mm and a length of 11.5 mm was implanted in 2019. The crown was installed in 2021. The screw worked for two years. The patient came for follow-up visits and the screw was tightened once.
9	lateral	single crown 46	IDI 3.7 mm × 12 mm	The broken screw attached a single crown to the implant in the area of the first mandibular premolar, right side (46) in the lateral section. Implant with a diameter of 3.7 mm and a length of 12 mm was implanted in 2009. The screw worked for one year. The patient did not come for follow-up visits.
10	lateral	single crown 36	ZIMMER 4.1 mm × 10 mm	Patient 10. The broken screw attached a single crown to the implant in the area of the first molar of the mandible, left side (36), lateral section. Implant with a diameter of 4.1 mm and a length of 10 mm was implanted in 2020. The crown was attached in 2020. The screw lasted three years and was tightened twice. The patient did not attend regular follow-up visits.

**Table 2:** Results of retrieved abutment screw investigation.

Case No.	Clinical Inspection	Optical/SEM Inspection	Lifetime Years
1	1 screws loosening	extremely numerous deep pits	4
2	2 fractured screws 2 screws loosening	extremely numerous deep pits	3
3	1 screws loosening	less numerous deep pits	2
4	1 screws loosening	numerous deep pits brownish sediment	8
5	1 screws loosening	numerous deep pits	7
6	1 screws loosening	moderately numerous deep pits	7
7	1 screws loosening	numerous deep pits white sediment	4
8	1 screws loosening	crack on the shank below head extremely numerous deep pits	2
9	1 fractured screw	few initial shallow pits difficult to see	1
10	1 fractured screw	numerous deep pits	3

## Data Availability

The original contributions presented in the study are included in the article, further inquiries can be directed to the corresponding author.
